# Editorial: Insights in animal behavior and welfare: 2021

**DOI:** 10.3389/fvets.2022.988463

**Published:** 2022-07-29

**Authors:** Edward Narayan

**Affiliations:** ^1^Faculty of Science, School of Agriculture and Food Sciences, The University of Queensland, St. Lucia, QLD, Australia; ^2^Centre for Animal Science, Queensland Alliance for Agriculture & Food Innovation, The University of Queensland, St. Lucia, QLD, Australia

**Keywords:** animal welfare, animal behavior, animal assisted interventions, animal therapy, stress, affective neuroscience

We are now entering the third decade of the twenty-first Century, and, especially in the last years, the achievements made by scientists have been exceptional, leading to major advancements in the fast-growing field of Animal Behavior and Welfare. In 2021, Frontiers organized a series of Research Topics to highlight the latest advancements in research across the field of Animal Behavior and Welfare, with articles from the members of our accomplished Editorial Board. This editorial initiative focusses on new insights, novel developments, current challenges, latest discoveries, recent advances, and future perspectives in the field of Animal Behavior and Welfare. The Research Topic solicited brief, forward-looking contributions from the editorial board members that describe the state of the art, outlining recent developments and major accomplishments that have been achieved and that need to occur to move the field forward.

The goal of this special edition Research Topic was to shed light on the progress made in the past decade in the Animal Behavior and Welfare field, and on its future challenges to provide a thorough overview of the field. This article collection will inspire, inform and provide direction and guidance to researchers in the field.

In 2021 Edition of this Topic, we show a collection of 6 peer reviewed articles which highlight the different advancements in the fields of animal welfare and behavior.

The first manuscript by Narayan et al. studied novel epigenetic markers, activity budget, physiological stress responses (wool cortisol) and wool quality of Merino sheep (*Ovis aries*) under single or twice annual shearing practice. Ewes managed under twice annual shearing expressed significantly lower levels of wool cortisol, 10% higher grazing activity and the lambs were born with better wool phenotype quality in terms of micron, spin fineness, and curvature. Novel epigenetic markers were discovered in the Merino ewes and lambs which can be evaluated further for improving genomic tools for sheep breeding and welfare programs.

In the second research, Sun et al. investigated the tongue rolling stereotypic behavior of Dairy Cows as an indicator of welfare. Researchers selected a cohort of 10 Holstein cows with or without tongue-rolling behavior and measured physical conditions, daily activity, rumen fermentation, and milk production. The results provided both physiological stress and metabolic differences in cows with or without tongue-rolling behavior. Cows with tongue-rolling behavior on average had high serum indicators of physiological stress. These cows also had higher energy metabolic status and they also showed more often drinking and lying behavior. The research provides baseline knowledge for further exploring the management of tongue-rolling behavior in dairy cows.

Three research articles were based on the welfare of dogs in various working environments, including animal-assisted interventions, police work, and dangerous fieldwork involving harmful chemicals.

Firstly, Miller et al. conducted a review of the welfare characteristics and temperament in working therapy dogs, with focus on positive affective state of the therapy dogs working in animal-assisted interventions. The researchers evaluated publications to determine the suitable biomarkers of the HPA axis, which can be used to evaluate positive welfare in therapy dogs. The review suggested that oxytocin could be used as an index of positive welfare as studies have shown that peripheral concentrations of oxytocin increases in dogs during positive social and affiliative interactions, including human-dog interaction. Aside from physiological and behavioral measures, the researchers also recommended that future studies of positive welfare assessments in therapy dogs should also consider the breed and temperament of the dog as influencing factors.

Gobbo and Šemrov conducted research on dogs to assess the self-control abilities under aggression reactivity. The study was based on police and privately owned dogs to study the associations between two aspects of inhibitory control in dogs, self-control, and cognitive inhibition. Police dogs showed higher aggression levels and poorer self-control than privately owned dogs, however no difference in cognitive inhibition. Researchers concluded that self-control or ability to tolerate delayed rewards, is key determinant of inhibitory control ability in police dogs.

Jarrett et al. carried out research on the working dogs that are exposed to dangerous work environments or harmful agent exposure. They evaluated the access to personal protective equipment and canine-specific field-use ready decontamination techniques and kits for use on working dogs, especially for exposure to harmful biologic or chemical agents.

Finally, in the sixth article in this Research Topic, Marchetti et al. evaluated the use of the international classification of diseases (ICD-11) method for veterinary forensic pathology for coding the cause and manner of death in wildlife. The research included the manner and the cause of death of 167 wild animals of 16 different species. Researchers concluded that the use of the ICD-11 method, as a sort of summary of the autopsy report, was confirmed to be of great value for the clarity and simplicity of processing the data collected also by veterinary pathologists. This tool has potential for future research to evaluate the human impact on wildlife in a scientific and statistically usable way.

Overall, this Topic highlights some the recent developments in the fields of animal welfare and behavior.

## Author contributions

The author confirms being the sole contributor of this work and has approved it for publication.

## Conflict of interest

The author declares that the research was conducted in the absence of any commercial or financial relationships that could be construed as a potential conflict of interest.

## Publisher's note

All claims expressed in this article are solely those of the authors and do not necessarily represent those of their affiliated organizations, or those of the publisher, the editors and the reviewers. Any product that may be evaluated in this article, or claim that may be made by its manufacturer, is not guaranteed or endorsed by the publisher.

